# National Guidelines for Cytoreductive Surgery and Hyperthermic Intraperitoneal Chemotherapy (HIPEC) in Peritoneal Malignancies: A Worldwide Systematic Review and Recommendations of Strength Analysis

**DOI:** 10.1245/s10434-025-17518-z

**Published:** 2025-05-24

**Authors:** Marco Tonello, Carola Cenzi, Elisa Pizzolato, Manuela Martini, Pierluigi Pilati, Antonio Sommariva

**Affiliations:** 1 Unit of Surgical Oncology of the Digestive Tract, Surgical Oncology Department, Veneto Institute of Oncology IOV-IRCCS, Padua, Castelfranco Veneto, TV Italy; 2 Clinical Research Unit, Veneto Institute of Oncology IOV-IRCCS, Padua, Italy

**Keywords:** Peritoneal malignancies, Cytoreductive surgery (CRS), HIPEC, National guidelines

## Abstract

**Background:**

National guidelines (GLs) for surgical cytoreduction (CRS) and hyperthermic intraperitoneal chemotherapy (HIPEC) in the management of peritoneal malignancies (PMs) vary across countries, scientific societies, and government agencies. This study aimed to systematically review and compare the recommendations for CRS/HIPEC in the treatment of ovarian cancer (EOC), gastric cancer, colorectal cancer (CRC), mesothelioma, and pseudomyxoma peritonei (PMP).

**Methods:**

Medical databases, search engines, and national websites of 193 countries were queried using artificial intelligence (AI)-powered software for scientific societies and/or government agencies guidelines. The study excluded consensus statements and guidelines without appropriate references. Non-English guidelines were translated, and data, including GRADE strength of recommendations, were extracted.

**Results:**

The study analyzed 138 guidelines, 24 for gastric cancer, 36 for colorectal cancer, 29 for primary ovarian cancer (p-)EOC, 28 for recurrent ovarian cancer (r-)EOC, 10 for mesothelioma, and 11 for PMP. Guidelines were retrieved from 51 (26.4%) nations, mostly from developed countries (62.1%; *p* < 0.001). The CRS procedure received robust positive recommendations (GRADE I/IIa) for CRC (74.2%), p-/r-EOC (100%/78.5%), PMP (90.9%), and mesothelioma (90.0%). Conversely, CRS was not indicated for gastric cancer (61.6%, GRADE III; *p* < 0.001). The HIPEC procedure had robust positive recommendations for PMP (90.9%) and mesothelioma (90.0%), but was controversial for p-EOC (42.3%) and CRC (38.0%) and contraindicated for r-EOC (80.0%) and gastric cancer (62.4%) (*p* < 0.001).

**Conclusion:**

National guidelines concordantly recommend CRS for colorectal cancer, ovarian cancer, PMP, and mesothelioma. In contrast, HIPEC recommendations are less homogeneously shared, except for PMP and mesothelioma. No positive concordance exists among guidelines on gastric cancer for CRS nor HIPEC. Furthermore, high-level evidence is needed to strengthen future guidelines on peritoneal metastases.

**Supplementary Information:**

The online version contains supplementary material available at 10.1245/s10434-025-17518-z.

Peritoneal malignancies (PMs) refer to involvement of the peritoneal surface by malignant tumor cells, either originating from the peritoneum itself (primary tumors) or spreading from other organs (peritoneal metastases). This condition is generally associated with a poor prognosis, significant impairment in quality of life, and obstructive symptoms. Whereas primary peritoneal tumors, such as serous carcinoma and mesothelioma, are rare, peritoneal metastases are common in epithelial ovarian cancer (60–70% of cases), gastric cancer (15–40%), pancreatic cancer (15%), and colorectal or appendiceal cancer (4–25%).^1^ Less frequently, metastases arise from biliary and genitourinary cancers or from extra-abdominal solid tumors such as breast and lung cancer.^2^

Before the 1990s, PMs were considered an incurable, end-stage disease with limited or no treatment options. However, with the introduction of multimodal therapy, including peritoneal surgery (i.e., cytoreductive surgery) and intraperitoneal chemotherapy, this paradigm has shifted in recent decades toward a potentially curative approach.^3^

Numerous reports from the 2000s, together with high-level studies in recent years, have demonstrated a significant improvement in survival among selected patients with PM of various origins.^4–6^ Based on these findings, several scientific societies and government agencies have incorporated cytoreductive surgery (CRS) and locoregional treatments, particularly hyperthermic intraperitoneal chemotherapy (HIPEC), into consensus statements and national guidelines. The strength of these recommendations varies depending on the type of tumor.

This study aimed to identify and analyze national guidelines worldwide regarding the recommendations for CRS and/or HIPEC in the treatment of pseudomyxoma peritonei (PMP), malignant peritoneal mesothelioma, and peritoneal malignancies with an epithelial ovarian, gastric, or colorectal origin.

## Methods

### Study Design

This study was designed to analyze and compare recommendations from national and international guidelines regarding indications CRS and/or HIPEC. The search focused on the most common malignancies associated with peritoneal metastases or primary peritoneal tumors, including high-grade serous ovarian carcinoma (epithelial ovarian cancer [EOC]), gastric cancer, colorectal cancer (CRC), PMP, and epithelial mesothelioma.

To be included in the analysis, the document had to meet the inclusion criteria requiring that they be published or endorsed by a national oncology or surgical oncology society with the stated goal of unconditionally improving cancer knowledge and treatment, be published or endorsed by an international oncology society or cancer alliance with a similar mission, be published by a government agency (e.g., national health ministry or equivalent), be published in English or the native language, provide sufficient evidence and references supporting the recommendation, explicitly or implicitly report the strength of the recommendation according to GRADE (Grading of Recommendations, Assessment, Development, and Evaluation),and be published within the last 20 years. Documents were excluded if they had consensus statements, expert opinions, insufficient references, or no evidence-based foundation, or had been published by organizations other than those specified in points 1, 2, or 3.

The literature and electronic source search, study design, and data analysis were performed in accordance with PRISMA (Preferred Reporting Items for Systematic Reviews and Meta-Analyses) guidelines.^7^ As a scoping review, the study was not registered with PROSPERO. References from relevant societies, government agencies, and collaborative groups were manually searched to identify additional potentially pertinent publications. Two researchers (M.T. and C.C.) independently selected studies based on the inclusion and exclusion criteria. Any discrepancies in study inclusion between the two researchers were resolved through discussion.

### Retrieval and Translation of National Guidelines

Two authors (M.T. and C.C.) conducted the search across various medical databases and search engines (e.g., MEDLINE, Scopus, Google Search, Google LLC) between 1 December and 20 December 2024. Artificial intelligence (AI)-powered softwares (ChatGPT by OpenAI LLC, USA and Perplexity AI, USA) were used for automated national guidelines searches, identification of the names and acronyms of national oncology and surgical oncology societies, and retrieval of the native language names and websites of health agencies and government bodies.

The search included the 193 sovereign countries that are members of the United Nations (UN), as listed on the UN website accessed in December 2024 Nations.^8^ The search strings used comprised the name of the nation, the primary tumor location, and a list of terms related to guidelines, as outlined in Supplementary Materials.

After an initial search, the authors observed an imbalance, with results predominantly from higher-income countries in Europe, North America, and Asia. To minimize bias in the visibility and retrieval of electronic resources from countries with lower economic income (primarily Africa, South America, and Oceania), the authors manually searched the websites of national oncology societies and health ministries from a sample of randomly selected countries. Six developing or least-developed countries, together with four additional countries, were included to represent all continents (list in Supplementary Materials). The list of developing and least-developed countries was obtained from the United Nations.^9^

All non-English guidelines were translated using at least two of the following software tools: DeepL (DeepL GmbH, Germany), MS Word (Microsoft Corp, Washington), and Google Translate (Google LLC, California). Critical terms for GRADE conversion (e.g., adjectives, verbal forms, and adverbs) were manually reviewed by two researchers (M.T. and C.C.) to ensure their relevance to the paragraph and statement meaning. For additional verification, a language-consulting service, the Veneto Institute of Oncology IOV-IRCCS, was provided by our institution.

### Data Extraction and Conversion to GRADE Recommendations

Data were extracted from the original documents using a pro-forma with predefined parameters, including country, primary tumor type, publication year, publisher (society, health agency, collaborative group/cancer alliance), reference to CRS, CRS (yes or no), reference to hyperthermic intraperitoneal chemotherapy (HIPEC), HIPEC (yes or no), and recommendation for CRS and/or HIPEC (positive, negative, not defined). Additionally, to compare the strength of recommendations across guidelines, the GRADE value (a systematic approach to rating the certainty of evidence) also was collected. Positive recommendations included GRADEs I, IIa, and IIb. Negative recommendations were classified as GRADE III, and “not defined” was used when the guidelines indicated insufficient data for any recommendation. For guidelines that did not report the GRADE value, the authors converted recommendations using the GRADE Working Group guidelines. Specifically, statements containing “is recommended,” “is indicated,” or “is effective” were considered as GRADE I (strong); whereas “is suggested,” “can be useful,” or “should be” were classified as GRADE IIa (moderate); “might be considered” or “might suggest” were classified as GRADE IIb (weak); and “do not recommend” or “is not beneficial” were classified as GRADE III (not to do).^10^ Any discrepancies between the two researchers were resolved through discussion between all authors or by adopting the most conservative approach (lower strength of recommendation).

### Statistical Analysis

Quantitative variables are presented as the median and interquartile range (IQR), whereas descriptive variables are expressed as counts and frequencies. Data were analyzed using the chi-square or Fisher’s exact test, as appropriate. Standardized residuals were used to identify subgroups that contributed most significantly to rejection of the null hypothesis, with a Bonferroni correction applied to adjust for *p* values.^11^ All statistical tests were two-sided, and *p* values lower than 0.05 were considered statistically significant. Statistical analyses were performed using SPSS software version 27 (SPSS Inc. Chicago, IL, USA).

## Results

### Records Selection

The study identified 1867 results through databases and gray literature searches. After removal of duplicates (*n* = 909), records published before 2014 (*n* = 121), and comments, letters, and errata (*n* = 51), 786 records remained for eligibility assessment.

**Fig. 1:**
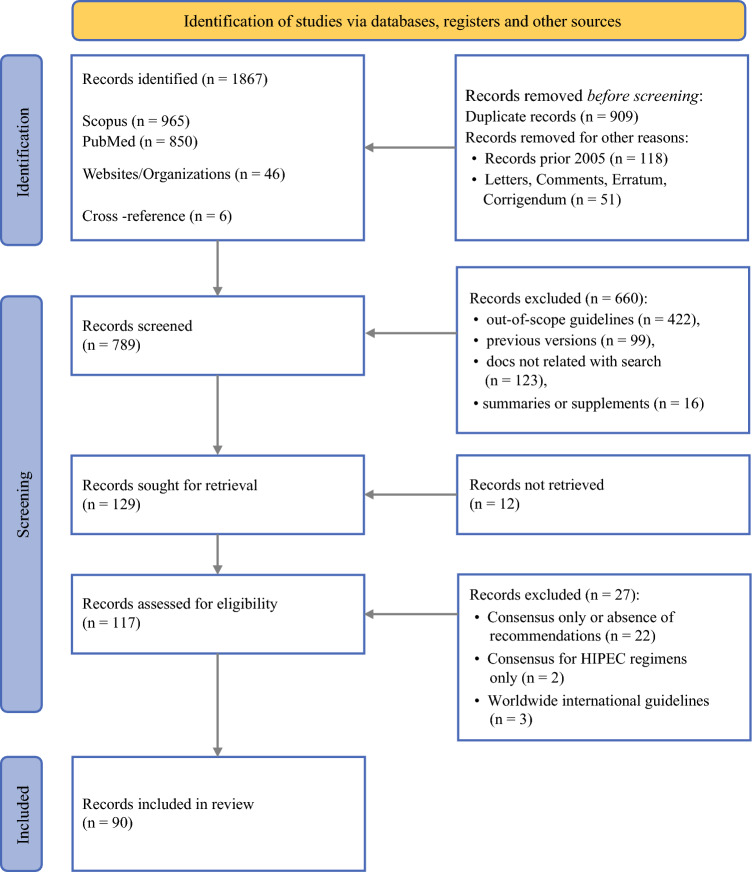
PRISMA flowchart of literature search. *Note*: records included also guidelines with multiple tumor types (e.g., GL on peritoneal malignancies were counted as five items because they addressed gastric, colorectal, primary, and recurrent ovarian cancer PM). PRISMA, Preferred Reporting Items for Systematic Reviews and Meta-Analyses; GL, national guidelines; PM, peritoneal malignancy

In the first round of screening (by title and abstract), 660 items were excluded because they were documents not related to guidelines or recommendations (*n* = 123), out-of-scope guidelines (*n* = 422), previous versions of the same guideline (*n* = 99), or summaries or supplements (*n* = 16). Of the remaining 126 records, 39 were removed because of inability to retrieve the full text (*n* = 12), consensus without recommendations or operative guidelines lacking appropriate references for indications (*n* = 22), consensus for HIPEC regimens only (*n* = 2), or worldwide multi-society international guidelines (*n* = 3). Ultimately, 87 records were included in the analysis (Fig. 1).

### General Characteristics of the Selected Guidelines

**Fig. 2:**
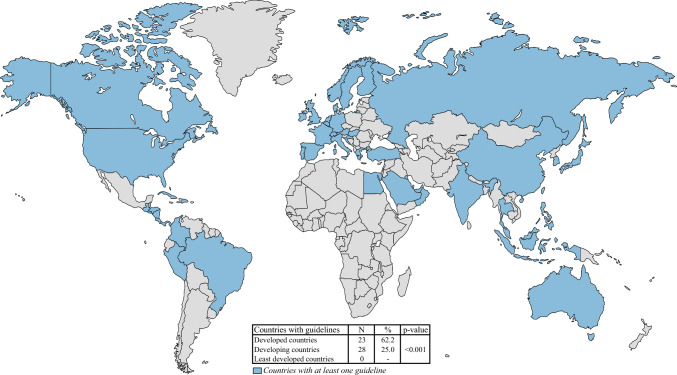
Countries with national guidelines

In the analysis, 138 guidelines were examined. Globally, 51 countries or international societies (representing 26.4% of the world's nations) have published guidelines for at least one of the investigated types of cancer, and 45 (88.2%) countries have more than one national guideline (GL) (Fig. 2). Among these, 23 developed countries (62.2%) have published or endorsed at least one guideline, compared with 25.0% of developing countries and none of the least-developed countries (*p* < 0.001).

Only six countries (3.1%) have guidelines on CRS and HIPEC for all the tumor types considered in this review, whereas the remaining countries lack guidelines for certain histologies, primarily malignant peritoneal mesothelioma and PMP. Most guidelines address peritoneal metastases in the section of advanced-stage disease (IIIc/IV) for specific cancer types (e.g., metastatic CRC). By contrast, five countries, representing 3.7% of the guidelines reviewed, have a single guideline addressing peritoneal malignancies from different origins. Of the guidelines reviewed, 81 (58.7%) were published by scientific societies, mainly oncologic societies (71.6%), and the remainder were promoted or endorsed by government or national agencies and cancer alliances (Table 1).

**Table 1:** Description of guidelines and recommendations

	GC	CRC	p-EOC	r-EOC	MM	PMP
	(*n* = 24) *n* (%)	(*n* = 36) *n* (%)	(*n* = 29) *n* (%)	(*n* = 28) *n* (%)	(*n* = 10) *n* (%)	(*n* = 11) *n* (%)
Median year (IQR)	2021 (2019–2024)	2022 (2018–2024)	2022 (2019–2024)	2021 (2019–2023)	2022 (2020–2022)	2022 (2020–2024)
Publisher						
Scientific society	18 (75.0)	19 (52.8)	17 (58.6)	16 (57.1)	5 (50.0)	6 (54.5)
Health agency/cancer alliance	6 (25.0)	17 (47.2)	12 (41.4)	12 (42.9)	5 (50.0)	5 (45.5)
CRS cited						
No	6 (25.0)	5 (13.9)	–	–	–	–
Yes	18 (75.0)	31 (86.1)	29 (100.0)	28 (100.0)	10 (100.0)	11 (100.0)
CRS indication						
Negative	11 (61.1)	–	–	1 (3.6)	1 (10.0)	1 (9.1)
Not defined	1 (5.6)	1 (3.2)	–	–	–	–
Positive	6 (33.3)	30 (96.8)	29 (100.0)	27 (96.4)	9 (90.0)	10 (90.9)
Inter-GL concordance, *p* value	**<0.001**					
HIPEC cited						
No	8 (33.3)	7 (19.4)	3 (10.3)	18 (64.3)	–	–
Yes	16 (66.7)	29 (80.6)	26 (89.7)	10 (35.7)	10 (100.0)	11 (100.0)
HIPEC indication						
Negative	10 (62.5)	7 (24.1)	10 (38.5)	8 (80.0)	1 (10.0)	1 (9.1)
Not defined	3 (18.8)	4 (13.8)	1 (3.8)	–	–	–
Positive	3 (18.8)	18 (62.1)	15 (57.7)	2 (20.0)	9 (90.0)	10 (90.9)
Inter-GL concordance, *p* value	**<0.001**					

Bold values indicate statistical signifcance (*p* < 0.05)

GC, gastric cancer; CRC, colorectal cancer; p-EOC, primary epithelial ovarian cancer; r-EOC, recurrent epithelial ovarian cancer; MM, malignant mesothelioma (epithelial); PMP, pseudomyxoma peritonei; IQR, interquartile range; CRS, cytoreductive surgery; GL, guidelines; HIPEC, intraperitoneal hyperthermic chemotherapy

The strength of recommendations was not reported by 38 (29.7%) guidelines, and data on GRADE were extracted from GL statements independently by two researchers (Fig. S1). Inter-researcher concordance was 90.8% and the seven cases of discrepancy were solved by discussion using the most conservative judgment (lower GRADE recommendation) (Tables S5 and S6). A dedicated table detailing the strength of recommendations for each histology across countries, together with the page numbers referencing CRS and HIPEC in the guidelines, can be found in the Supplementary Materials (Tables S1 and S2).

### Peritoneal Metastases From Gastric Cancer (GC)

**Fig. 3:**
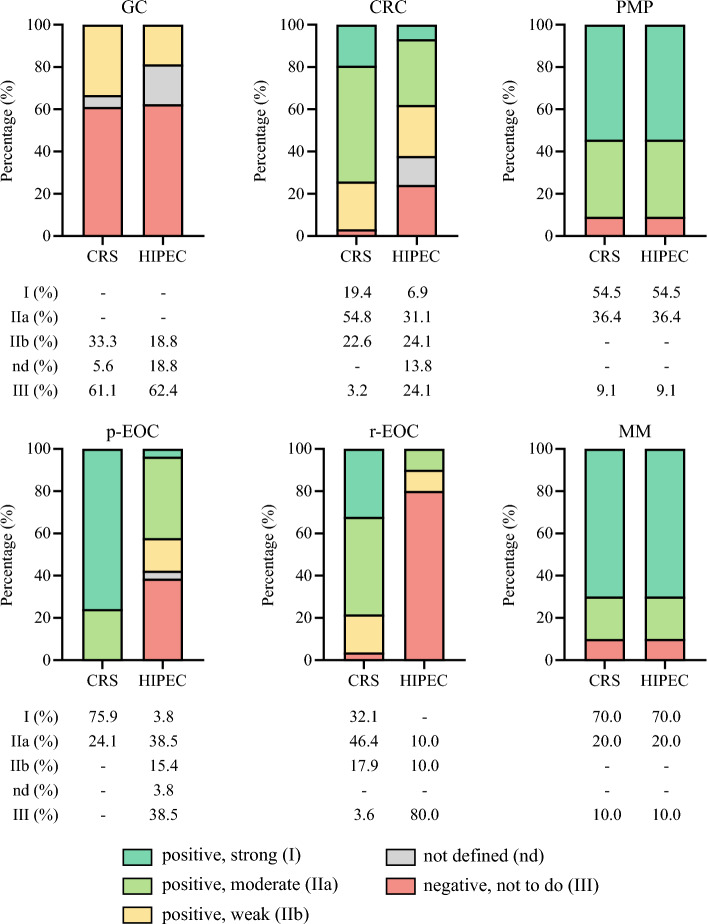
Recommended GRADE in guidelines. GRADE, Grading of Recommendations, Assessment, Development, and Evaluation; GC, gastric cancer; CRC, colorectal cancer; p-EOC, primary epithelial ovarian cancer; r-EOC, recurrent epithelial ovarian cancer; MM, malignant mesothelioma (epithelial); PMP, pseudomyxoma peritonei; CRS, cytoreductive surgery; HIPEC, intraperitoneal hyperthermic chemotherapy.

Of the retrieved guidelines, 24 addressed the treatment of peritoneal metastases from gastric cancer.^12–33^ The median publication year was 2021 (IQR, 2019–2024), and 75% of the guidelines were published by scientific societies. Cytoreductive surgery was mentioned in 18 guidelines (75.0%), with negative recommendations (GRADE III, not to perform CRS) in 11 guidelines (61.1%). Six guidelines provided positive indications for CRS, although with weak strength (GRADE IIb). The HIPEC procedure was cited in 16 guidelines (66.7%), 10 (62.5%) of which had a negative recommendation (GRADE III, not to perform HIPEC) (Table 1, Fig. 3).

### Peritoneal Metastases From CRC

The study analyzed 36 guidelines addressing peritoneal metastases from CRC.^14,22,34–65^ Half of these guidelines (52.8%) were published by scientific societies in 2022 (IQR, 2018–2024). Cytoreductive surgery was cited in 31 guidelines (86.1%), with 30 (96.8%) showing strong agreement for positive indications, albeit with varying strengths of recommendation (19.4% GRADE I, 54.8% GRADE IIa, and 22.6% GRADE IIb). The HIPEC procedure was discussed in 29 guidelines (80.6%) and recommended in 18 cases (62.1%), with differing strengths (6.9% GRADE I, 31.1% GRADE IIa, and 24.1% GRADE IIb) (Table 1; Fig. 3).

### Peritoneal Metastases From Primary and Recurrent Epithelial Ovarian Cancer (p-EOC and r-EOC)

The analysis focused on high-grade serous ovarian carcinoma (HGSOC), retrieving 29 guidelines for the primary treatment of peritoneal metastases (p-EOC) and 28 guidelines for recurrent peritoneal metastases (r-EOC).^14,20,22,52,66–89^ The guidelines were published primarily by scientific societies (57.9%) in 2022 (IQR, 2019–2024). Cytoreductive surgery was recommended in both primary and recurrent settings, with a GRADE I/IIa endorsement of 100% in p-EOC guidelines and 78.4% in r-EOC guidelines, and with specific selection criteria for recurrent patients, such as platinum sensitivity. In most guidelines, HIPEC was cited for primary EOC (89.7%) and recommended in 15 guidelines (57.7%), with moderate strength in 38.5%. Most guidelines indicated CRS-HIPEC only in the interval setting (after neoadjuvant chemotherapy). In the recurrent setting, HIPEC was cited in only 35.7% of guidelines, with a negative recommendation in most cases (80.0% GRADE III; Table 1, Fig. 3).

### Malignant Peritoneal Mesothelioma (MM)

Guidelines for managing malignant peritoneal mesothelioma (MM, epithelial subtype) are less common than those for other tumors. In some cases, MM is briefly mentioned in recommendations for pleural mesothelioma, lacking specific therapeutic pathways. After eligibility assessment, only 10 guidelines addressing the treatment of MM were indeed identified and included in this review.^14,22,90–95^ Half of these were published primarily by scientific societies and international collaborative groups in 2022 (IQR, 2020–2022). Except for one, all guidelines considered CRS and HIPEC as standard treatments (GRADE I; Table 1, Fig. 3).

### PMP

Due to the rarity of the condition, guidelines for PMP also are scarce. The study selected 11 guidelines, with 54.5% published by scientific societies in 2022 (IQR, 2020–2024).^14,22,54,61,65,90,91,96,97^ In some cases, recommendations for PMP were included in the CRC guidelines (Table S2, Supplementary Materials). Similar to mesothelioma, all but one guideline considered CRS and HIPEC as standard treatments for PMP (GRADE I; Table 1, Fig. 3).

### Guidelines Agreement

When the concordance of recommendations was compared across specific tumor types, significant differences were observed for both CRS (*p* < 0.001) and HIPEC (*p* < 0.001). Post hoc subgroup analysis using standardized residuals showed that only the recommendations for CRS in gastric cancer exhibited a high disagreement (33.3% positive, 5.6% not defined, 61.1% negative; *p* < 0.001). Regarding HIPEC, recommendations were largely concordant only for recurrent EOC (80% negative; *p* = 0.001), mesothelioma (90.0% positive; *p* < 0.001), and PMP (90.9% positive; *p* < 0.001) (Table S3; Supplementary Materials).

## Discussion

The introduction of surgical treatment for peritoneal metastases has improved survival rates for selected patients across various tumor types, including CRC, PMP, peritoneal mesothelioma, and epithelial ovarian cancer.^98–101^ The combination of CRS and HIPEC has been proposed as a promising multimodal approach to address microscopic tumor residuals after complete surgery.^102^

As with any new technique, the CRS and HIPEC procedures were initially met with a mix of enthusiasm and skepticism, largely due to the lack of robust evidence. During the past decades, a significant body of lower-level evidence has been gathered, particularly on CRC in Europe and gastric cancer in East Asia. This has prompted the scientific community to design more rigorous trials despite the challenges of enrolling patients with peritoneal malignancies in surgical studies. Currently, some high-level evidence exists, and in recent years, many scientific societies and government agencies have begun including CRS-HIPEC in national guidelines. This systematic review was designed to analyze the recommendations for CRS and/or HIPEC in the national guidelines across all countries for selected peritoneal malignancies published in the last 10 years.

Globally, 51 countries (26.1%) have at least one guideline on peritoneal malignancies. Most of the 138 guidelines analyzed are published by high-income countries (62.2%; *p* = 0.001), likely reflecting the considerable costs as well as the ancillary organizational and infrastructural requirements of CRS-HIPEC. Actually, no guidelines were retrieved by least developed countries, probably due to the prohibitive expenses and requirements of the procedure. Interestingly, only five countries (2.6%) provide guidelines addressing peritoneal malignancies from multiple origins, whereas the remainder discuss peritoneal metastases within the advanced disease section of the guidelines for specific tumor types.

The concordance of recommendations across guidelines generally mirrors the level of available evidence, as seen in CRC and primary epithelial ovarian carcinoma. On the contrary, PMP and mesothelioma demonstrate nearly complete consensus despite the lack of randomized controlled trials. Indeed, a comparison of guideline concordance across specific tumors shows significant differences among groups (*p* < 0.001, chi-square). Post hoc subgroup analysis demonstrates that agreement among guidelines is very low for CRS in gastric cancer and for HIPEC in CRC, primary EOC, and gastric cancer.

High-level evidence regarding the treatment of peritoneal metastases from gastric cancer remains limited. Currently, no randomized controlled trials have assessed the efficacy of CRS, and existing studies on HIPEC yield conflicting results. One trial reported a survival benefit of HIPEC compared with CRS alone, although with a small sample size.^103^ By contrast, the GASTRIPEC trial found no overall advantage.^104^ However, a subanalysis of the latter study suggested improved survival when HIPEC was added for patients with no residual disease after CRS. This limited and inconsistent evidence is reflected in the predominantly negative or undefined recommendations (66.7% for CRS, 81.2% for HIPEC) across the 24 guidelines that were evaluated.

In CRC, outside the prophylactic or adjuvant setting, two phase 3 trials have investigated CRS and HIPEC. The first study compared CRS-HIPEC with systemic therapy and demonstrated a survival benefit in the surgical and locoregional treatment arm, leading to increased recognition of CRS-HIPEC in guidelines.^105^ The more recent PRODIGE7 trial evaluated the addition of oxaliplatin-based HIPEC to CRS, but found no survival advantage, reinforcing the role of CRS in colorectal peritoneal metastases while raising debate about the efficacy of HIPEC.^98^ These findings are reflected in the varied levels of positive recommendations across analyzed guidelines, with 96.8% endorsing CRS and 62.1% supporting HIPEC. Some guidelines also acknowledge the ongoing scientific debate on the optimal HIPEC regimen for colorectal peritoneal metastases, specifying GRADE III (not to do) recommendations exclusively for oxaliplatin-based HIPEC while not extending this classification to other agents such as mitomycin C.^65^

For peritoneal metastases from high-grade serous epithelial ovarian carcinoma, CRS and HIPEC can be proposed at various stages of the disease, either at diagnosis (primary setting) or upon recurrence after primary surgery. In the primary setting, CRS-HIPEC can be performed as an upfront procedure before systemic chemotherapy, as an interval surgery after three cycles of a carboplatin-taxol regimen or after the completion of systemic treatment, typically after six cycles (total neoadjuvant therapy). The role of CRS in EOC, specifically with achievement of no residual disease, is well-established and widely accepted, supported by the findings of the DESKTOP III trial.^99^ Conversely, the role of HIPEC remains a topic of debate. The OVHIPEC study demonstrated a survival benefit when HIPEC was added to CRS as an interval treatment.^106^ A similar finding was observed in a subgroup analysis of a Korean randomized controlled trial.^107^ Additionally, a Spanish phase 3 study reported a survival advantage for HIPEC after neoadjuvant systemic chemotherapy.^108^ Currently,evidence to support upfront CRS-HIPEC is insufficient, as the only study on this approach yielded negative results.^107^

The results of our study reflect the widespread acceptance of CRS in the management of advanced EOC. All guidelines provide a positive endorsement for CRS, with 75.9% of cases receiving a GRADE I recommendation. On the other hand, despite high-level evidence supporting interval HIPEC, many guidelines express reservations, resulting in more controversial findings (57.7% positive support with a moderate [GRADE IIa] recommendation in 38.5% of cases).

In the recurrent setting, two randomized trials demonstrated a survival benefit for platinum-sensitive patients treated with CRS, but found no advantage in adding HIPEC.^109,110^ Of the 28 guidelines analyzed, all but one recommended CRS for these patients, with 46.4% assigning a moderate GRADE IIa recommendation. In contrast, only 10 guidelines included a positive recommendation for HIPEC, with only two providing positive endorsements (20.0%).

Pseudomyxoma peritonei and malignant peritoneal mesothelioma are rare conditions, and high-level studies on their management are lacking. Current recommendations are primarily based on large clinical series and expert consensus.^100,101,111,112^ Considering the rarity of these conditions, international consortia and prospective databases are more likely to be the source of robust evidence than randomized trials. Despite this, nearly complete concordance exists among the analyzed guidelines, with all but one recommending CRS and HIPEC as the standard treatment (GRADE I/IIa recommendation: PMP 90.9% and MM 90.0%). This consensus likely stems from promising survival outcomes and the lack of effective systemic therapy options, especially for PMP.

Discordance between clinical guidelines, in some cases even within the same country, can lead to variations in clinical practice. Decisions regarding CRS and/or HIPEC are often influenced by the policies of individual oncologic centers and the specialized expertise of their surgical or oncologic teams. To improve adherence to guidelines, it is essential to foster global collaboration among oncologic and surgical societies as well as health agencies. This collaboration should aim to support high-quality international studies on common malignancies and establish registries for rarer tumors.

The limitations of the current study primarily arose from biases in the selection and interpretation of guidelines, which are influenced by search tools and translation from native languages. Although searching of electronic medical databases is relatively standardized, the vast body of information in gray literature, such as government publications, reports, and conference proceedings, poses a challenge for analysis without the help of search engines or AI-powered software. The algorithms behind these tools are proprietary and inaccessible to users, potentially introducing bias in the retrieved records. Consequently, some published guidelines may have been overlooked in our analysis. Furthermore, given that CRS-HIPEC is a costly procedure requiring advanced health care infrastructure, most guidelines are concentrated in developed countries of the Northern Hemisphere. We attempted to mitigate this bias by manually searching for guidelines from developing countries, least developed countries, and nations in the Southern Hemisphere.

Another concern is the issue of linguistic interpretation and translation. Because a minority of the included guidelines did not explicitly report the GRADE strength of recommendations, we converted guideline statements into recommendations using the GRADE Working Group’s criteria in which specific adverbs and sentences have a suggested translation into recommendations (e.g., “is indicated” = GRADE I, “can be useful” = GRADE IIa, “might reasonable” = GRADE IIb). Because this process was prone to interpretation errors, to minimize biases, two researchers independently evaluated the guidelines and derived a GRADE recommendation with a low inter-observer variability (9.2%). In cases of uncertainty, the most conservative (lower strength) recommendation was adopted. Also the translation process could have introduced errors because non-English-speaking authors may have translated their native terms with approximation, and AI-powered translation tools may have misinterpreted nuances. To minimize this potential bias, we used at least two different translation software tools, carefully reviewing full sentences to ensure that they aligned with the intended meaning of the recommendation.

## Conclusion

Of the 51 countries with at least one guideline on peritoneal malignancies, only 11.8% cover all the tumor types discussed in this review. Recommendations for CRS are positive and concordant for CRC, epithelial ovarian carcinoma, PMP, and peritoneal mesothelioma, whereas HIPEC is clearly and homogeneously recommended in the reviewed guidelines only for PMP and mesothelioma. For gastric cancer, CRS and HIPEC are debated, with negative recommendations in the majority of guidelines. Further high-level evidence is needed to harmonize and strengthen national guidelines for the treatment of peritoneal metastases.

## Supplementary Information

Below is the link to the electronic supplementary material.

Supplementary file1 (DOCX 43 KB)
