# Supplementary material for: National Guidelines for Cytoreductive Surgery and Hyperthermic Intraperitoneal Chemotherapy (HIPEC) in Peritoneal Malignancies: A Worldwide Systematic Review and Recommendations of Strength Analysis
Source: Ann Surg Oncol. 2025 May 24;32(8):5795–806. doi: 10.1245/s10434-025-17518-z (PMC12222385; doi:10.1245/s10434-025-17518-z)
Supplement: Supplementary file 1 — Supplementary file1 (DOCX 43 KB) [file 10434_2025_17518_MOESM1_ESM.docx]

**Table S1. National guidelines and strength of recommendations (GRADE).**

|  | **GC** | | **CRC** | | **p-EOC** | | **r-EOC**** | | **MM** | | **PMP** | |
| --- | --- | --- | --- | --- | --- | --- | --- | --- | --- | --- | --- | --- |
|  | **CRS** | **HIPEC** | **CRS** | **HIPEC** | **CRS** | **HIPEC** | **CRS** | **HIPEC** | **CRS** | **HIPEC** | **CRS** | **HIPEC** |
| **Australia** | **–** | **–** | **IIb** | **IIb** | **–** | **–** | **–** | **–** | **–** | **–** | **–** | **–** |
| **Austria** | **III** | **III** | **I** | **?*** | **I** | **III** | **IIa** | **●** | **I** | **I** | **I** | **I** |
| **Belgium** | **–** | **–** | **IIa** | **IIb** | **IIa** | **IIa** | **I** | **III** | **–** | **–** | **–** | **–** |
| **Brazil** | **III** | **III** | **–** | **–** | **IIa** | **IIa** | **IIa** | **●** | **I** | **I** | **I** | **I** |
| **CAC** | **–** | **–** | **IIb** | **IIb** | **–** | **–** | **–** | **–** | **–** | **–** | **–** | **–** |
| **Canada** | **III** | **III** | **III** | **III** | **I** | **IIa** | **III** | **III** | **III** | **III** | **III** | **III** |
| **China** | **IIb** | **?** | **●** | **●** | **–** | **–** | **–** | **–** | **–** | **–** | **–** | **–** |
| **Colombia** | **–** | **–** | **–** | **–** | **IIa** | **III** | **IIa** | **III** | **–** | **–** | **–** | **–** |
| **Croatia** | **–** | **–** | **IIa** | **IIa** | **I** | **III** | **IIb** | **III** | **–** | **–** | **–** | **–** |
| **Denmark** | **●** | **●** | **IIb** | **IIb** | **IIa** | **III** | **IIb** | **●** | **–** | **–** | **–** | **–** |
| **Egypt** | **–** | **–** | **IIa** | **III** | **–** | **–** | **–** | **–** | **–** | **–** | **–** | **–** |
| **Europe** | **IIb** | **?** | **IIa** | **III** | **I** | **?** | **IIb** | **III** | **I** | **I** | **I** | **I** |
| **Finland** | **–** | **–** | **I** | **I** | **–** | **–** | **–** | **–** | **–** | **–** | **–** | **–** |
| **France** | **–** | **–** | **IIb** | **IIb** | **I** | **IIa** | **I** | **IIa** | **–** | **–** | **–** | **–** |
| **France** | **IIb** | **IIb** | **IIa** | **IIa** | **I** | **IIa** | **I** | **●** | **–** | **–** | **IIa** | **IIa** |
| **GCC** | **–** | **–** | **IIa** | **●** | **–** | **–** | **–** | **–** | **–** | **–** | **–** | **–** |
| **Germany** | **III** | **III** | **I** | **?*** | **I** | **III** | **IIa** | **●** | **I** | **I** | **I** | **I** |
| **Greece** | **III** | **III** | **IIa** | **IIa** | **–** | **–** | **–** | **–** | **–** | **–** | **–** | **–** |
| **Hungary** | **–** | **–** | **–** | **–** | **IIa** | **III** | **IIb** | **III** | **–** | **–** | **–** | **–** |
| **India** | **●** | **●** | **IIa** | **IIb** | **I** | **IIb** | **I** | **●** | **–** | **–** | **–** | **–** |
| **Ireland** | **–** | **–** | **●** | **●** | **–** | **–** | **–** | **–** | **–** | **–** | **–** | **–** |
| **Italy** | **?** | **?** | **IIa** | **III** | **I** | **●** | **I** | **III** | **–** | **–** | **–** | **–** |
| **Italy** | **III** | **III** | **IIa** | **IIa** | **IIa** | **IIa** | **IIa** | **●** | **IIa** | **IIa** | **IIa** | **IIa** |
| **Japan** | **●** | **●** | **IIa** | **●** | **I** | **III** | **IIa** | **●** | **–** | **–** | **–** | **–** |
| **Malaysia** | **–** | **–** | **IIb** | **IIb** | **IIa** | **IIb** | **IIa** | **IIb** | **–** | **–** | **–** | **–** |
| **Netherlands** | **III** | **III** | **I** | **I*** | **I** | **I** | **I** | **●** | **I** | **I** | **–** | **–** |
| **Norway** | **●** | **●** | **IIa** | **IIa** | **I** | **●** | **IIa** | **●** | **–** | **–** | **IIa** | **IIa** |
| **Pan-Asian** | **III** | **III** | **I** | **III** | **–** | **–** | **–** | **–** | **–** | **–** | **–** | **–** |
| **Peru** | **–** | **–** | **●** | **●** | **–** | **–** | **–** | **–** | **–** | **–** | **–** | **–** |
| **Portugal** | **–** | **–** | **–** | **–** | **I** | **IIb** | **IIa** | **●** | **–** | **–** | **–** | **–** |
| **Russia** | **III** | **III** | **●** | **●** | **I** | **●** | **I** | **●** | **IIa** | **IIa** | **–** | **–** |
| **Saudi Arabia** | **●** | **●** | **●** | **●** | **I** | **IIa** | **IIa** | **●** | **–** | **–** | **–** | **–** |
| **South Korea** | **IIb** | **●** | **IIa** | **IIa** | **I** | **IIa** | **I** | **●** | **–** | **–** | **–** | **–** |
| **Spain** | **III** | **●** | **IIb** | **III** | **I** | **III** | **I** | **III** | **–** | **–** | **–** | **–** |
| **Sweden** | **●** | **●** | **IIa** | **IIa** | **I** | **III** | **IIa** | **●** | **–** | **–** | **I** | **I** |
| **Switzerland** | **III** | **III** | **I** | **?*** | **I** | **III** | **IIa** | **●** | **I** | **I** | **I** | **I** |
| **Turkey** | **IIb** | **IIb** | **–** | **–** | **–** | **–** | **–** | **–** | **–** | **–** | **–** | **–** |
| **UK** | **–** | **–** | **IIa** | **IIa** | **I** | **IIb** | **IIb** | **●** | **–** | **–** | **–** | **–** |
| **USA (ASCRS)** | **–** | **–** | **IIa** | **IIa** | **–** | **–** | **–** | **–** | **–** | **–** | **–** | **–** |
| **USA (ASCO)** | **–** | **–** | **IIb** | **III** | **I** | **IIa** | **–** | **–** | **–** | **–** | **–** | **–** |
| **USA (NCCN)** | **IIb** | **IIb** | **IIa** | **?*** | **I** | **IIa** | **IIa** | **●** | **I** | **I** | **IIa** | **IIa** |

*Abbreviations*. GC, gastric cancer; CRC, colorectal cancer; p-EOC, primary epithelial ovarian cancer; r-EOC, recurrent epithelial ovarian cancer; MM, malignant mesothelioma (epithelial); PMP, pseudomyxoma peritonei; CRS, cytoreductive surgery; HIPEC, hyperthermic intraperitoneal chemotherapy; CAC, central america and caribbean; GCC, gulf cooperation countries. GRADE I (strong); IIa (moderate); IIb (weak); GRADE III (not to do); **?** not defined; **●**: not cited in guidelines; **–** no guideline retrieved for the country/society/collaborative group; *: guidelines for HIPEC in CRC consider drug regimen (Oxaliplatin and Mitomicin C); **: r-EOC guidelines recommend CRS+/-HIPEC only in Platinum-sensitive patients.

**Table S2. References to national guidelines addressing CRS and/or HIPEC.**

|  | **GC** | **CRC** | **p-EOC** | **r-EOC** | **MM** | **PMP** |
| --- | --- | --- | --- | --- | --- | --- |
| **Australia** | – | [37], *p.87* | – | – | – | – |
| **Austria** | [12], *p.29* | [35], *p.29* | [66], *p.14* | [66], *p.17* | [90]*, p.15* | [90]*, p.16* |
| **Belgium** | – | [36], *p.9* | [67], *p.14-16* | [67], *p.16* | – | – |
| **Brazil** | [13], *p.6* | – | [68], *p.1246* | [68], *p.1249* | [91], *p.530* | [91], *p.530* |
| **CAC** | – | [38] | – | – | – | – |
| **Canada** | [14], *p. 9* | [14], *p. 8* | [14], *p. 3* | [14], *p. 5* | [14], *p. 11* | [14], *p. 12* |
| **China** | [15], *p.150* | [39] | – | – | – | – |
| **Colombia** | – | – | [69], *p.129* | [69], *p.129* | – | – |
| **Croatia** | – | [40] | [70], *p.421* | [70], *p.129* | – | – |
| **Denmark** | [16] | [41] | [71] | [71] | – | – |
| **Egypt** | – | [42] | – | – | – | – |
| **Europe** | [17], *p.1008* | [43]*, p.17* | [72], *p.255* | [72], *p.258* | [92]*, p.20* | [96], *p.20* |
| **Finland** | – | [44] | – | – | – | – |
| **France** | – | [45], *p.9* | [73], *p.9* | [73], *p.9* | – | – |
| **France** | [18], *p.772* | [46] | [74, 75] | [74, 75] | – | [97] |
| **GCC** | – | [34], *p.10* | – | – | – | – |
| **Germany** | [12], *p.29* | [35], *p.29* | [66], *p.14* | [66], *p.17* | [90]*, p.15* | [90]*, p.16* |
| **Greece** | [19] | [47] | – | – | – | – |
| **Hungary** | – | – | [76] | [76] | – | – |
| **India** | [20] | [48] | [20] | [20] | – | – |
| **Ireland** | – | [49] | – | – | – | – |
| **Italy** | [21] | [50]*, p.154* | [77], *p.25* | [77], *p.27* | – | – |
| **Italy** | [22], *p.138* | [22], *p.131* | [22], *p.107* | [22], *p.112* | [22], *p.96* | [22], *p.76* |
| **Japan** | [23] | [51]*, p.49* | [78] | [78] | – | – |
| **Malaysia** | – | [52]*, p.36* | [52]*, p.10* | [52]*, p.10* | – | – |
| **Netherlands** | [24], *p.190* | [53]*, p.68* | [79], *p.68* | [79] | [93]*, p.77* | – |
| **Norway** | [25] | [54] | [80] | [80] | – | [54] |
| **Pan-Asian** | [26], *p.22* | [55]*, p.3* | – | – | – | – |
| **Peru** | – | [56] | – | – | – | – |
| **Portugal** | – | – | [81], *p.76* | [81], *p.84* | – | – |
| **Russia** | [27] | [57] | [82] | [82] | [94] | – |
| **Saudi Arabia** | [28] | [58] | [83]*, p.8* | [83]*, p.11* | – | – |
| **South Korea** | [29] | [59], *p.8* | [84]*, p.2* | [84]*, p.6* | – | – |
| **Spain** | [30], *p.10* | [60], *p.2722* | [85]*, p.6* | [85]*, p.6* | – | – |
| **Sweden** | [31] | [61], *p.111* | [86]*, p.72* | [86] | – | [61], *p.87* |
| **Switzerland** | [12], *p.29* | [35], *p.29* | [66], *p.14* | [66], *p.17* | [90]*, p.15* | [90]*, p.16* |
| **Turkey** | [32], *p. 539* | – | – | – | – | – |
| **UK** | – | [62], *p.21* | [87] | [87] | – | – |
| **USA (ASCRS)** | – | [63] | – | – | – | – |
| **USA (ASCO)** | – | [64], *p.688* | [88] | – | – | – |
| **USA (NCCN)** | [33], *p. 7* | [65], *p.103* | [89]*, p.35* | [89], *p.36* | [95], *p.16* | [65], *p.61* |

References to documents are reported in squared brackets. *Abbreviations*. *p.*, guideline page addressing CRS and/or HIPEC; GC, gastric cancer; CRC, colorectal cancer; p-EOC, primary epithelial ovarian cancer; r-EOC, recurrent epithelial ovarian cancer; MM, malignant mesothelioma (epithelial); PMP, pseudomyxoma peritonei; CRS, cytoreductive surgery; HIPEC, hyperthermic intraperitoneal chemotherapy; CAC, central america and caribbean; GCC, gulf cooperation countries.

**Table S3. Post-hoc analysis of concordance between guidelines.**

|  | **CRS** | | | | | | | | | |
| --- | --- | --- | --- | --- | --- | --- | --- | --- | --- | --- |
|  | *Adjusted residuals* | | | | | *p values* | | | | |
|  | **ND** | **III** | **IIb** | **IIa** | **I** | **ND** | **III** | **IIb** | **IIa** | **I** |
| **GC** | 3.414 | 6.781 | 2.380 | -3.360 | -3.782 | **0.000640** | **0.000000** | 0.017313 | **0.000779** | **0.000156** |
| **CRC** | -0.806 | -1.689 | 1.567 | 2.877 | -2.584 | 0.420243 | 0.091219 | 0.117115 | 0.004015 | 0.009766 |
| **p-EOC** | -0.771 | -2.231 | -2.477 | -1.226 | 4.619 | 0.440707 | 0.025681 | 0.013249 | 0.220199 | **0.000004** |
| **r-EOC** | -0.754 | -1.516 | 0.653 | 1.627 | -0.849 | 0.450849 | 0.129519 | 0.513756 | 0.103737 | 0.395881 |
| **MM** | -0.415 | -0.176 | -1.332 | -0.948 | 2.088 | 0.678142 | 0.860294 | 0.182860 | 0.343129 | 0.036798 |
| **PMP** | -0.437 | -0.283 | -1.403 | 0.203 | 1.101 | 0.662111 | 0.777177 | 0.160617 | 0.839135 | 0.270897 |

|  | **HIPEC** | | | | | | | | | |
| --- | --- | --- | --- | --- | --- | --- | --- | --- | --- | --- |
|  | *Adjusted residuals* | | | | | *p values* | | | | |
|  | **ND** | **III** | **IIb** | **IIa** | **I** | **ND** | **III** | **IIb** | **IIa** | **I** |
| **GC** | 2.364 | 2.154 | 0.395 | -2.622 | -1.935 | 0.018079 | 0.031240 | 0.692843 | 0.008742 | 0.052990 |
| **CRC** | 1.137 | -1.561 | 1.725 | 0.847 | -1.515 | 0.255538 | 0.118524 | 0.084527 | 0.396995 | 0.129772 |
| **p-EOC** | -1.022 | 0.312 | 0.137 | 1.795 | -1.903 | 0.306781 | 0.755041 | 0.891031 | 0.072654 | 0.057041 |
| **r-EOC** | -1.030 | 3.057 | -0.431 | -1.168 | -1.427 | 0.303010 | **0.001118** | 0.666468 | 0.242807 | 0.153580 |
| **MM** | -1.030 | -1.798 | -1.374 | -0.402 | 5.004 | 0.303010 | 0.072177 | 0.169442 | 0.687684 | **0.000001** |
| **PMP** | -1.086 | -1.963 | -1.449 | 0.898 | 3.779 | 0.277479 | 0.049646 | 0.147338 | 0.369186 | **0.000157** |

Inter-GL concordance was calculated on post-hoc adjusted residuals comparing differences in recommendation between guidelines concordance. In bold significant values after Bonferroni correction for 30 iterations (p_BON_ < 0.001667). In bold significant results using corrected p value. Abbreviations: GC gastric cancer, CRC colorectal cancer, p-EOC primary epithelial ovarian cancer, r-EOC recurrent epithelial ovarian cancer, MM malignant mesothelioma (epithelial), PMP pseudomyxoma peritonei, CRS cytoreductive surgery, HIPEC intraperitoneal hyperthermic chemotherapy

**Table S5. Inter-researcher metrics for strength of recommendations (GRADE) of Cytoreductive surgery.**

| **CRS** | **GC** | | **CRC** | | **p-EOC** | | **r-EOC** | | **MM** | | **PMP** | |
| --- | --- | --- | --- | --- | --- | --- | --- | --- | --- | --- | --- | --- |
|  | **CC** | **MT** | **CC** | **MT** | **CC** | **MT** | **CC** | **MT** | **CC** | **MT** | **CC** | **MT** |
| **Australia*** | **–** | **–** | **IIb** | **IIb** | **–** | **–** | **–** | **–** | **–** | **–** | **–** | **–** |
| **Austria** | **III** | **III** | **I** | **I** | **I** | **I** | **IIa** | **IIa** | **I** | **I** | **I** | **I** |
| **Belgium** | **–** | **–** | **IIa** | **IIa** | **IIa** | **IIa** | **I** | **I** | **–** | **–** | **–** | **–** |
| **Brazil** | **III** | **III** | **–** | **–** | **IIa** | **IIa** | **IIa** | **IIa** | **I** | **I** | **I** | **I** |
| **CAC** | **–** | **–** | **IIb** | **IIb** | **–** | **–** | **–** | **–** | **–** | **–** | **–** | **–** |
| **Canada*** | **III** | **III** | **III** | **III** | **I** | **I** | **III** | **III** | **III** | **III** | **III** | **III** |
| **China** | **IIb** | **IIb** | **●** | **●** | **–** | **–** | **–** | **–** | **–** | **–** | **–** | **–** |
| **Colombia** | **–** | **–** | **–** | **–** | **IIa** | **IIa** | **IIa** | **IIa** | **–** | **–** | **–** | **–** |
| **Croatia** | **–** | **–** | **IIa** | **IIa** | **I** | **I** | **IIb** | **IIb** | **–** | **–** | **–** | **–** |
| **Denmark** | **●** | **●** | **IIb** | **IIb** | **IIa** | **IIa** | **IIb** | **IIb** | **–** | **–** | **–** | **–** |
| **Egypt** | **–** | **–** | **IIa** | **IIa** | **–** | **–** | **–** | **–** | **–** | **–** | **–** | **–** |
| **Europe*** | **IIa** | **IIb** | **IIa** | **IIa** | **I** | **I** | **IIb** | **IIb** | **I** | **I** | **I** | **I** |
| **Finland** | **–** | **–** | **I** | **I** | **–** | **–** | **–** | **–** | **–** | **–** | **–** | **–** |
| **France** | **–** | **–** | **IIb** | **IIb** | **I** | **I** | **I** | **I** | **–** | **–** | **–** | **–** |
| **France** | **IIb** | **IIb** | **IIa** | **IIa** | **I** | **I** | **I** | **I** | **–** | **–** | **IIa** | **IIa** |
| **GCC** | **–** | **–** | **IIa** | **IIa** | **–** | **–** | **–** | **–** | **–** | **–** | **–** | **–** |
| **Germany*** | **III** | **III** | **I** | **I** | **I** | **I** | **IIa** | **IIa** | **I** | **I** | **I** | **I** |
| **Greece** | **III** | **III** | **IIa** | **IIa** | **–** | **–** | **–** | **–** | **–** | **–** | **–** | **–** |
| **Hungary*** | **–** | **–** | **–** | **–** | **IIa** | **IIa** | **IIa** | **IIb** | **–** | **–** | **–** | **–** |
| **India** | **●** | **●** | **IIa** | **IIa** | **I** | **I** | **I** | **I** | **–** | **–** | **–** | **–** |
| **Ireland** | **–** | **–** | **●** | **●** | **–** | **–** | **–** | **–** | **–** | **–** | **–** | **–** |
| **Italy** | **?** | **?** | **IIa** | **IIa** | **I** | **I** | **I** | **I** | **–** | **–** | **–** | **–** |
| **Italy** | **III** | **III** | **IIa** | **IIa** | **IIa** | **IIa** | **IIa** | **IIa** | **IIa** | **IIa** | **IIa** | **IIa** |
| **Japan** | **●** | **●** | **IIa** | **IIa** | **I** | **I** | **IIa** | **IIa** | **–** | **–** | **–** | **–** |
| **Malaysia*** | **–** | **–** | **IIb** | **IIb** | **IIa** | **IIa** | **IIa** | **IIa** | **–** | **–** | **–** | **–** |
| **Netherlands*** | **III** | **III** | **I** | **I** | **I** | **I** | **I** | **I** | **I** | **I** | **–** | **–** |
| **Norway*** | **●** | **●** | **IIa** | **IIa** | **I** | **I** | **IIa** | **IIa** | **–** | **–** | **IIa** | **IIa** |
| **Pan-Asian** | **III** | **III** | **I** | **I** | **–** | **–** | **–** | **–** | **–** | **–** | **–** | **–** |
| **Peru** | **–** | **–** | **●** | **●** | **–** | **–** | **–** | **–** | **–** | **–** | **–** | **–** |
| **Portugal*** | **–** | **–** | **–** | **–** | **I** | **I** | **IIa** | **IIa** | **–** | **–** | **–** | **–** |
| **Russia** | **III** | **III** | **●** | **●** | **I** | **I** | **I** | **I** | **IIa** | **IIa** | **–** | **–** |
| **Saudi Arabia** | **●** | **●** | **●** | **●** | **I** | **I** | **IIa** | **IIa** | **–** | **–** | **–** | **–** |
| **South Korea** | **IIb** | **IIb** | **IIa** | **IIa** | **I** | **I** | **I** | **I** | **–** | **–** | **–** | **–** |
| **Spain** | **III** | **III** | **IIb** | **IIb** | **I** | **I** | **I** | **I** | **–** | **–** | **–** | **–** |
| **Sweden** | **●** | **●** | **IIa** | **IIa** | **I** | **I** | **IIa** | **IIa** | **–** | **–** | **I** | **I** |
| **Switzerland*** | **III** | **III** | **I** | **I** | **I** | **I** | **IIa** | **IIa** | **I** | **I** | **I** | **I** |
| **Turkey*** | **IIb** | **IIb** | **–** | **–** | **–** | **–** | **–** | **–** | **–** | **–** | **–** | **–** |
| **UK*** | **–** | **–** | **IIa** | **IIa** | **I** | **I** | **IIb** | **IIb** | **–** | **–** | **–** | **–** |
| **USA (ASCRS)** | **–** | **–** | **IIa** | **IIa** | **–** | **–** | **–** | **–** | **–** | **–** | **–** | **–** |
| **USA (ASCO)** | **–** | **–** | **IIb** | **IIb** | **I** | **I** | **–** | **–** | **–** | **–** | **–** | **–** |
| **USA (NCCN)** | **IIb** | **IIb** | **IIa** | **IIa** | **I** | **I** | **IIa** | **IIa** | **I** | **I** | **IIa** | **IIa** |

*Abbreviations*. GC, gastric cancer; CRC, colorectal cancer; p-EOC, primary epithelial ovarian cancer; r-EOC, recurrent epithelial ovarian cancer; MM, malignant mesothelioma (epithelial); PMP, pseudomyxoma peritonei; CRS, cytoreductive surgery; HIPEC, hyperthermic intraperitoneal chemotherapy; CAC, central america and caribbean; GCC, gulf cooperation countries. GRADE I (strong); IIa (moderate); IIb (weak); GRADE III (not to do); **?** not defined; **●**: not cited in guidelines; **–** no guideline retrieved for the country/society/collaborative group; *: GRADE recommendation derived by researchers (the remainders were explicitly reported); for Europe only in GC, for UK only in CRC; blue shaded cells: discordance between researchers.

**Table S6. Inter-researcher metrics for strength of recommendations (GRADE) of HIPEC.**

| **HIPEC** | **GC** | | **CRC** | | **p-EOC** | | **r-EOC** | | **MM** | | **PMP** | |
| --- | --- | --- | --- | --- | --- | --- | --- | --- | --- | --- | --- | --- |
|  | **CC** | **MT** | **CC** | **MT** | **CC** | **MT** | **CC** | **MT** | **CC** | **MT** | **CC** | **MT** |
| **Australia*** | **–** | **–** | **IIb** | **IIb** | **–** | **–** | **–** | **–** | **–** | **–** | **–** | **–** |
| **Austria** | **III** | **III** | **?** | **?** | **III** | **III** | **●** | **●** | **I** | **I** | **I** | **I** |
| **Belgium** | **–** | **–** | **IIb** | **IIb** | **IIa** | **IIa** | **III** | **III** | **–** | **–** | **–** | **–** |
| **Brazil** | **III** | **III** | **–** | **–** | **IIa** | **IIa** | **●** | **●** | **I** | **I** | **I** | **I** |
| **CAC** | **–** | **–** | **IIb** | **IIb** | **–** | **–** | **–** | **–** | **–** | **–** | **–** | **–** |
| **Canada*** | **III** | **III** | **III** | **III** | **IIa** | **IIa** | **III** | **III** | **III** | **III** | **III** | **III** |
| **China** | **?** | **?** | **●** | **●** | **–** | **–** | **–** | **–** | **–** | **–** | **–** | **–** |
| **Colombia** | **–** | **–** | **–** | **–** | **III** | **III** | **III** | **III** | **–** | **–** | **–** | **–** |
| **Croatia** | **–** | **–** | **IIa** | **IIa** | **III** | **III** | **III** | **III** | **–** | **–** | **–** | **–** |
| **Denmark** | **●** | **●** | **IIb** | **IIb** | **III** | **III** | **●** | **●** | **–** | **–** | **–** | **–** |
| **Egypt** | **–** | **–** | **III** | **III** | **–** | **–** | **–** | **–** | **–** | **–** | **–** | **–** |
| **Europe*** | **?** | **?** | **III** | **III** | **?** | **?** | **III** | **III** | **I** | **I** | **I** | **I** |
| **Finland** | **–** | **–** | **I** | **I** | **–** | **–** | **–** | **–** | **–** | **–** | **–** | **–** |
| **France** | **–** | **–** | **IIb** | **IIb** | **IIa** | **IIa** | **IIa** | **IIa** | **–** | **–** | **–** | **–** |
| **France** | **IIb** | **IIb** | **IIa** | **IIa** | **IIa** | **IIa** | **●** | **●** | **–** | **–** | **IIa** | **IIa** |
| **GCC** | **–** | **–** | **●** | **●** | **–** | **–** | **–** | **–** | **–** | **–** | **–** | **–** |
| **Germany*** | **III** | **III** | **?** | **?** | **III** | **III** | **●** | **●** | **I** | **I** | **I** | **I** |
| **Greece** | **III** | **III** | **IIa** | **IIa** | **–** | **–** | **–** | **–** | **–** | **–** | **–** | **–** |
| **Hungary*** | **–** | **–** | **–** | **–** | **III** | **III** | **III** | **III** | **–** | **–** | **–** | **–** |
| **India** | **●** | **●** | **IIb** | **IIb** | **IIb** | **IIb** | **●** | **●** | **–** | **–** | **–** | **–** |
| **Ireland** | **–** | **–** | **●** | **●** | **–** | **–** | **–** | **–** | **–** | **–** | **–** | **–** |
| **Italy** | **?** | **?** | **III** | **III** | **●** | **●** | **III** | **III** | **–** | **–** | **–** | **–** |
| **Italy** | **III** | **III** | **IIa** | **IIa** | **IIa** | **IIa** | **●** | **●** | **IIa** | **IIa** | **IIa** | **IIa** |
| **Japan** | **●** | **●** | **●** | **●** | **III** | **III** | **●** | **●** | **–** | **–** | **–** | **–** |
| **Malaysia*** | **–** | **–** | **IIb** | **IIa** | **IIb** | **IIa** | **IIb** | **IIa** | **–** | **–** | **–** | **–** |
| **Netherlands*** | **III** | **III** | **I** | **I** | **I** | **I** | **●** | **●** | **I** | **I** | **–** | **–** |
| **Norway*** | **●** | **●** | **IIa** | **IIa** | **●** | **●** | **●** | **●** | **–** | **–** | **IIa** | **IIa** |
| **Pan-Asian** | **III** | **III** | **III** | **III** | **–** | **–** | **–** | **–** | **–** | **–** | **–** | **–** |
| **Peru** | **–** | **–** | **●** | **●** | **–** | **–** | **–** | **–** | **–** | **–** | **–** | **–** |
| **Portugal*** | **–** | **–** | **–** | **–** | **IIa** | **IIb** | **●** | **●** | **–** | **–** | **–** | **–** |
| **Russia** | **III** | **III** | **●** | **●** | **●** | **●** | **●** | **●** | **IIa** | **IIa** | **–** | **–** |
| **Saudi Arabia** | **●** | **●** | **●** | **●** | **IIa** | **IIa** | **●** | **●** | **–** | **–** | **–** | **–** |
| **South Korea** | **●** | **●** | **IIa** | **IIa** | **IIa** | **IIa** | **●** | **●** | **–** | **–** | **–** | **–** |
| **Spain** | **●** | **●** | **III** | **III** | **III** | **III** | **III** | **III** | **–** | **–** | **–** | **–** |
| **Sweden** | **●** | **●** | **IIa** | **IIa** | **III** | **III** | **●** | **●** | **–** | **–** | **I** | **I** |
| **Switzerland*** | **III** | **III** | **?** | **?** | **III** | **III** | **●** | **●** | **I** | **I** | **I** | **I** |
| **Turkey*** | **IIa** | **IIb** | **–** | **–** | **–** | **–** | **–** | **–** | **–** | **–** | **–** | **–** |
| **UK*** | **–** | **–** | **IIa** | **IIa** | **IIb** | **IIb** | **●** | **●** | **–** | **–** | **–** | **–** |
| **USA (ASCRS)** | **–** | **–** | **IIa** | **IIa** | **–** | **–** | **–** | **–** | **–** | **–** | **–** | **–** |
| **USA (ASCO)** | **–** | **–** | **III** | **III** | **IIa** | **IIa** | **–** | **–** | **–** | **–** | **–** | **–** |
| **USA (NCCN)** | **IIb** | **IIb** | **?** | **?** | **IIa** | **IIa** | **●** | **●** | **I** | **I** | **IIa** | **IIa** |

*Abbreviations*. GC, gastric cancer; CRC, colorectal cancer; p-EOC, primary epithelial ovarian cancer; r-EOC, recurrent epithelial ovarian cancer; MM, malignant mesothelioma (epithelial); PMP, pseudomyxoma peritonei; CRS, cytoreductive surgery; HIPEC, hyperthermic intraperitoneal chemotherapy; CAC, central america and caribbean; GCC, gulf cooperation countries. GRADE I (strong); IIa (moderate); IIb (weak); GRADE III (not to do); **?** not defined; **●**: not cited in guidelines; **–** no guideline retrieved for the country/society/collaborative group; *: GRADE recommendation derived by researchers (the remainders were explicitly reported); for Europe only in GC, for UK only in CRC; blue shaded cells: discordance between researchers.

**Search strategy**

**1). Search strings for Scopus and Pubmed**

**String 1 -** *( TITLE ( consensus OR guideline* OR recommendation* ) AND TITLE ( colorectal OR ovarian OR ovary OR colon OR gastric OR pseudomyxoma OR "peritoneal mesothelioma" ) AND TITLE ( treatment OR management ) )*

**String 2 -** *( TITLE ( consensus OR guideline* OR recommendation* ) AND TITLE ( periton* ) AND TITLE ( metastas* OR malignancies OR carcinomatosis ) AND TITLE ( treatment OR management ) )*

**String 3 -** *( TITLE ( consensus OR guideline* OR recommendation* ) AND TITLE ( hipec ) )*

**2). ChatGPT and Perplexity AI search**

Provide the names of [CONTINENT]'s societies of medical oncology. Report the names of societies for all countries in [CONTINENT]. Moreover, provide links to their websites.

Provide the names of [CONTINENT]'s societies of surgical oncology. Report the names of societies for all countries in [CONTINENT]. Moreover, provide links to their websites.

Provide the names of [CONTINENT]'s health agencies. Report the names of health agencies or health ministries for all countries in [CONTINENT]. Moreover, provide links to their websites.

Provide links to [COUNTRY]’s national guidelines for oncological treatment.

In square brackets it was manually added the name of the continent/country for each search round.

**3). List of developed, developing, and least-developed countries and manually checked using world wide web search engines (e.g Google Inc.) for guidelines and health agencies (randomly chosen to cover low–income countries and all continents):**

1. Albania
2. Algeria
3. Argentina
4. Australia
5. Brazil
6. Chile
7. Egypt
8. Israel
9. Laos
10. Libya
11. Mexico
12. Morocco
13. New Zealand
14. Rwanda
15. Russia
16. Serbia
17. South Africa
18. Tunisia
19. Ukraine
20. Venezuela
